# Pernicious Propaganda

**Published:** 1918-04-20

**Authors:** 


### AN IMPERIAL UNION OF NURSES.

### Pernicious Propaganda.

The champions of faction in the British nursing world are alive to the importance of propaganda, and are conducting a vigorous campaign in favour of disunion in America and the Colonies. It is interesting in this connection to note that Editors of prominent nursing journals, like *Una*, published in Melbourne, stand aloof from expressing any opinion. It is a wise attitude until the issues are made plain. The arguments presented in that paper by Miss Eden are prolix and highly technical, and convey the impression that a vulgar family squabble is in progress here, the wider issues being completely obscured.

In order to understand the situation it is necessary to think in a large way. To enter into the maze of petty and futile argument that is put forward is to get lost. The broad facts in a few words are these. For a period of thirty years, up to the outbreak of the war, certain people, mainly ladies, interested themselves in the cause of British nurses. Let us be generous, and admit high motive and hard work. During all this time, the British nurse, like the British soldier, occupied an obscure position in the public eye, with the result that very little was achieved. Miss Rimmer, in her very concise statement in *Una*, sums up this achievement in a single sentence. "The 1914 Bill," says Miss Rimmer, "is the considered result of thirty years' work for nurses." This is a perfectly accurate and also a perfectly complete statement. Nothing more and nothing less than the drawing up of a Parliamentary Bill stands to the credit of the early pioneers, and I am glad to have this succinctly stated by one of themselves because it saves much argument and demonstration.

Now the College of Nursing is the offspring of the war. The British soldier and the British nurse, allies in the conflict, have suddenly sprung into prominence, so that the humblest citizen realises that both one and other of them are as necessary to the preserving of the existence of the Empire as the guns of the British fleet. The College of Nursing is the embodiment of a great and far-reaching ideal—the union and betterment of nurses not only in Great Britain but throughout the Empire. It is as though the soul of Florence Nightingale had come back to inspire the British people to complete for the members of the profession the noble work which she began. To suggest that such an ideal can be reached by means of an Act of Parliament is to show an utter incapacity to grasp the situation. An Imperial Union of Nurses, backed by a grateful Empire, can accomplish marvels, and the psychological moment is now, while the tide is flowing. Miss Eden, in *Una*, advises nurses to study the Bills. "It is the Bills that matter—read them and arrange to have them explained. That is the only right way." This advice is not in the least degree likely to be followed, but if it is it will add nothing to the barren story of which Miss Rimmer boasts. For the uplifting, benefiting, and, if you like, the glorifying of the nurses of the Empire two factors are necessary and two only—a complete union of themselves and generous public support. A Parliamentary Bill may be a useful incident but nothing more, and it is failure to appreciate this fact that is responsible for the thirty unprogressive years. Like the fishermen of Galilee they toiled all night and caught nothing.

The question arises, why did these good people not join forces with the College? The explanation is that they failed to understand its import. They mistook it for a smaller craft and determined to sink it. It is melancholy to record, but the fact remains, that the interests of nurses were deliberately sacrificed lest their own personalities should be submerged in an all-embracing National and Imperial Union. For thirty years, lean though they were, it had been a case of "we are the people, and when we speak let no dog bark." Note the predominant note of their objections. It is not a case of British nurses and the British public, but of "us" and "our societies." The British public are described as "gold-bugs," "employers of labour," "society women," "laymen," and so forth, and the individual nurse is appealed to to be "independent" and to despise "charity." Does any sane man or woman regard the nation's tribute of £44,000 to Florence Nightingale as "charity"? Does any sane man or woman regard the British Red Cross as a charitable institution for the British soldier? It is nothing of the sort, but, instead, the civilian effort of the Empire to win the war. The appeal to the nation on behalf of the British nurse, and its response, is a branch of this civilian effort, and then women after a lifetime of futile endeavour are leaving no stone unturned to ruin the movement, and thereby prevent the nurses from reaping the fruits of their labours. *Vide* Lavinia Dock in the *Amercan Journal of Nursing*: "That odious element, combined of patronising, charity-mongering, undemocratic bossism, which has been the affliction of British nurses for thirty years, is still busy trying to enslave them in a web wherein the College of Nursing, State Registration, and public alms are woven with the intent to keep them professionally helpless." Such a tirade of misrepresentation is the obvious product of wounded vanity. It is abuse, not argument.

However we may feel sorry for the zealous advocates of a day that is dead, it is futile to cling to principles and schemes that are obsolete. In the nursing world, as elsewhere, the chariot of progress rides along, and those who elect to lag behind must be reconciled to shed their tears in vain. Miss Eden informs the Editor of *Una* that "hundreds of nurses are joining [the College] with misgivings in their hearts." They will not long have misgivings. It is the nurses' hour, and the larger the number (9,000 already) who take advantage of a golden opportunity the richer will be the ultimate harvest.

